# Supplementary material for: MALDI-MS Analysis of Peptide Libraries Expands the Scope of Substrates for Farnesyltransferase
Source: Int J Mol Sci. 2021 Nov 7;22(21):12042. doi: 10.3390/ijms222112042 (PMC8584866; doi:10.3390/ijms222112042)
Supplement: Supplementary file 1 [file ijms-22-12042-s001.zip › ijms-1425708-supplementary.pdf]

MALDI-MS analysis of peptide libraries expands the scope of substrates for farnesyltransferase

Garrett L Schey[1], Peter H Buttery[2], Emily R Hildebrandt[3], Sadie X Novak[4], Walter K Schmidt[3], James L Hougland[4,5], Mark D Distefano[1,2]

1 Department of Medicinal Chemistry, University of Minnesota, Minneapolis, Minnesota 55455, USA
2 Department of Chemistry, University of Minnesota, Minneapolis, Minnesota 55455, USA
3 Department of Biochemistry and Molecular Biology, University of Georgia, Athens, Georgia 30602, USA
4 Department of Chemistry, Syracuse University, Syracuse, New York 13244 USA
5 BioInspired Syracuse, Syracuse University, Syracuse, New York 13244 USA

Table of Contents

Table S1. Mass of CaaaX library peptides 3
Figure S1. Optimization of enzyme concentration for CaaaX MALDI 6
Figure S2. MALDI/MS of CMIIX Library 7
Figure S3. MALDI/MS of CMa2IM Library 8
Figure S4. MALDI/MS of CMIa3M Library 9
Table S2. MS/MS of prenylated CaaaX library hits 10
Figure S5. Structure of representative DsGRAGC(fn)MIIM peptide 12
Figure S6. HPLC Fluorescence assay of CMIIS 12
Figure S7. HPLC Fluorescence assay of CSIIM 13
Figure S8. HPLC Fluorescence assay of CMKIM....................13
Figure S9. HPLC Fluorescence assay of CMIGM.................... 14
Figure S10. HPLC Fluorescence assay of CHIIM 14
Figure S11. HPLC Fluorescence assay of CMIIK 15
Figure S12. HPLC Fluorescence assay of CYIIM 15
Table S3. CaaaX sequences in the human genome 16

Figure S13. Mobility shift analysis of Ydj1p-CaaaX variants in the presence and absence of farnesyltransferase 17
Table S4. Percent farnesylation for Ydj1p-CaaaX variants evaluated in this study 17
Figure S14. Mobility shift analysis of Ydj1p-CaaaX variants 18
Table S5. Yeast strains used in this study 19
Table S6. Plasmids used in this study 19

Library	Peptide Sequence	Expected Mass	Observed Mass
Ca$_1$IIM 1	DsGRAGCGIIM	1110.4	1110.2
Ca$_1$IIM 1	DsGRAGCSIIM	1140.4	1140.2
Ca$_1$IIM 1	DsGRAGC(fn)SIIM	1344.8	1344.4
Ca$_1$IIM 1	DsGRAGCVIIM	1152.5	1152.3
Ca$_1$IIM 1	DsGRAGCCIIM	1156.5	1156.2
Ca$_1$IIM 1	DsGRAGC(fn)CIIM	1360.8	1360.4
Ca$_1$IIM 1	DsGRAGCIIIM	1166.5	1166.3
Ca$_1$IIM 1	DsGRAGCNIIM	1167.4	1167.3
Ca$_1$IIM 1	DsGRAGC(fn)NIIM	1371.8	1371.4
Ca$_1$IIM 1	DsGRAGCKIIM	1181.5	1181.3
Ca$_1$IIM 1	DsGRAGCMIIM	1184.5	1184.2
Ca$_1$IIM 1	DsGRAGC(fn)MIIM	1388.9	1388.4
Ca$_1$IIM 1	DsGRAGCFIIM	1200.5	1200.3
Ca$_1$IIM 1	DsGRAGC(fn)FIIM	1404.9	1404.4
Ca$_1$IIM 1	DsGRAGCYIIM	1216.5	1216.2
Ca$_1$IIM 1	DsGRAGC(fn)YIIM	1420.9	1420.4
Ca$_1$IIM 2	DsGRAGCAIIM	1124.4	1124.3
Ca$_1$IIM 2	DsGRAGC(fn)AIIM	1328.8	1328.4
Ca$_1$IIM 2	DsGRAGCPIIM	1150.4	1150.3
Ca$_1$IIM 2	DsGRAGC(fn)PIIM	1354.8	1354.4
Ca$_1$IIM 2	DsGRAGCTIIM	1154.4	1154.2
Ca$_1$IIM 2	DsGRAGCLIIM	1166.5	1166.3
Ca$_1$IIM 2	DsGRAGCDIIM	1168.4	1168.2
Ca$_1$IIM 2	DsGRAGCQIIM	1181.5	1181.2
Ca$_1$IIM 2	DsGRAGC(fn)QIIM	1385.8	1385.4
Ca$_1$IIM 2	DsGRAGCEIIM	1182.4	1182.3
Ca$_1$IIM 2	DsGRAGC(fn)EIIM	1386.8	1386.4
Ca$_1$IIM 2	DsGRAGCHIIM	1190.5	1190.3
Ca$_1$IIM 2	DsGRAGC(fn)HIIM	1394.8	1394.4
Ca$_1$IIM 2	DsGRAGCRIIM	1209.5	1209.3
Ca$_1$IIM 2	DsGRAGCWIIM	1239.5	1239.3
CMa$_2$IM 1	DsGRAGCMGIM	1128.4	1128.1
CMa$_2$IM 1	DsGRAGC(fn)MGIM	1332.8	1332.2
CMa$_2$IM 1	DsGRAGCMSIM	1158.4	1158.1
CMa$_2$IM 1	DsGRAGC(fn)MSIM	1362.8	1362.3
CMa$_2$IM 1	DsGRAGCMVIM	1170.5	1170.1
CMa$_2$IM 1	DsGRAGCMCIM	1174.5	1174.1
CMa$_2$IM 1	DsGRAGCMIIM	1184.5	1184.2
CMa$_2$IM 1	DsGRAGCMNIM	1185.5	1185.1
CMa$_2$IM 1	DsGRAGC(fn)MNIM	1389.2	1389.3
CMa$_2$IM 1	DsGRAGCMKIM	1199.5	1199.2
CMa$_2$IM 1	DsGRAGC(fn)MKIM	1403.9	1303.4
CMa$_2$IM 1	DsGRAGCMMIM	1202.6	1202.1
CMa$_2$IM 1	DsGRAGCMFIM	1218.5	1218.1
CMa$_2$IM 1	DsGRAGCMYIM	1234.5	1234.1
CMa$_2$IM 2	DsGRAGCMAIM	1142.4	1142.1
CMa$_2$IM 2	DsGRAGCMPIM	1168.5	1168.1
CMa$_2$IM 2	DsGRAGCMTIM	1172.5	1172.1

CMa$_2$IM 2	DsGRAGCMLIM	1184.5	1184.1
CMa$_2$IM 2	DsGRAGCMDIM	1186.5	1186.1
CMa$_2$IM 2	DsGRAGCMQIM	1199.5	1199.1
CMa$_2$IM 2	DsGRAGC(fn)MQIM	1403.9	1403.3
CMa$_2$IM 2	DsGRAGCMEIM	1200.5	1200.1
CMa$_2$IM 2	DsGRAGC(fn)MEIM	1404.8	1404.3
CMa$_2$IM 2	DsGRAGCMHIM	1208.5	1208.1
CMa$_2$IM 2	DsGRAGC(fn)MHIM	1412.9	1412.3
CMa$_2$IM 2	DsGRAGCMRIM	1227.6	1227.1
CMa$_2$IM 2	DsGRAGC(fn)MRIM	1431.9	1431.3
CMa$_2$IM 2	DsGRAGCMWIM	1257.6	1257.1
CMIa$_3$M 1	DsGRAGCMIGM	1128.4	1128.2
CMIa$_3$M 1	DsGRAGC(fn)MIGM	1332.8	1332.3
CMIa$_3$M 1	DsGRAGCMISM	1158.4	1158.2
CMIa$_3$M 1	DsGRAGC(fn)MISM	1362.8	1362.3
CMIa$_3$M 1	DsGRAGCMIVM	1170.5	1170.3
CMIa$_3$M 1	DsGRAGCMICM	1174.5	1174.2
CMIa$_3$M 1	DsGRAGC(fn)MICM	1378.9	1378.3
CMIa$_3$M 1	DsGRAGCMIIM	1184.5	1184.3
CMIa$_3$M 1	DsGRAGCMINM	1185.5	1185.2
CMIa$_3$M 1	DsGRAGC(fn)MINM	1389.2	1389.3
CMIa$_3$M 1	DsGRAGCMIKM	1199.5	1199.3
CMIa$_3$M 1	DsGRAGCMIMM	1202.6	1202.2
CMIa$_3$M 1	DsGRAGC(fn)MIMM	1406.9	1406.3
CMIa$_3$M 1	DsGRAGCMIFM	1218.5	1218.2
CMIa$_3$M 1	DsGRAGC(fn)MIFM	1422.9	1422.3
CMIa$_3$M 1	DsGRAGCMIYM	1234.5	1234.2
CMIa$_3$M 2	DsGRAGCMIAM	1142.4	1142.3
CMIa$_3$M 2	DsGRAGC(fn)MIAM	1346.8	1346.4
CMIa$_3$M 2	DsGRAGCMIPM	1168.5	1168.3
CMIa$_3$M 2	DsGRAGCMITM	1172.5	1172.4
CMIa$_3$M 2	DsGRAGC(fn)MITM	1376.8	1376.5
CMIa$_3$M 2	DsGRAGCMILM	1184.5	1184.3
CMIa$_3$M 2	DsGRAGC(fn)MILM	1388.9	1388.5
CMIa$_3$M 2	DsGRAGCMIDM	1186.5	1186.4
CMIa$_3$M 2	DsGRAGCMIQM	1199.5	1199.4
CMIa$_3$M 2	DsGRAGC(fn)MIQM	1403.9	1403.5
CMIa$_3$M 2	DsGRAGCMIEM	1200.5	1200.3
CMIa$_3$M 2	DsGRAGC(fn)MIEM	1404.8	1404.5
CMIa$_3$M 2	DsGRAGCMIHM	1208.5	1208.4
CMIa$_3$M 2	DsGRAGC(fn)MIHM	1412.9	1412.5
CMIa$_3$M 2	DsGRAGCMIRM	1227.6	1227.4
CMIa$_3$M 2	DsGRAGCMIWM	1257.6	1257.4
CMIIX 1	DsGRAGCMIIG	1110.4	1110.3
CMIIX 1	DsGRAGCMIIS	1140.4	1140.3
CMIIX 1	DsGRAGC(fn)MIIS	1344.8	1344.4
CMIIX 1	DsGRAGCMIIV	1152.5	1152.3
CMIIX 1	DsGRAGCMIIC	1156.5	1156.3
CMIIX 1	DsGRAGC(fn)MIIC	1360.8	1360.4

CMIIX 1	DsGRAGCMIII	1166.5	1166.3
CMIIX 1	DsGRAGCMIIN	1167.4	1167.2
CMIIX 1	DsGRAGCMIIK	1181.5	1181.3
CMIIX 1	DsGRAGC(fn)MIIK	1385.9	1385.5
CMIIX 2	DsGRAGCMIIA	1124.4	1124.2
CMIIX 2	DsGRAGC(fn)MIIA	1328.8	1328.4
CMIIX 2	DsGRAGCMIIP	1150.4	1150.3
CMIIX 2	DsGRAGCMIIT	1154.4	1154.2
CMIIX 2	DsGRAGCMIIL	1166.5	1166.3
CMIIX 2	DsGRAGCMIID	1168.4	1168.2
CMIIX 2	DsGRAGCMIIQ	1181.5	1181.3
CMIIX 2	DsGRAGC(fn)MIIQ	1385.8	1385.4
CMIIX 2	DsGRAGCMIIM	1184.5	1184.2
CMIIX 2	DsGRAGC(fn)MIIM	1388.9	1388.4
CMIIX 2	DsGRAGCMIIR	1209.5	1209.3
CMIIX 2	DsGRAGCMIIW	1239.5	1239.3

Table S1. Mass of CaaaX library peptides. Expected and observed mass for all pentapeptide starting material from MALDI libraries, as well as the prenylated products.

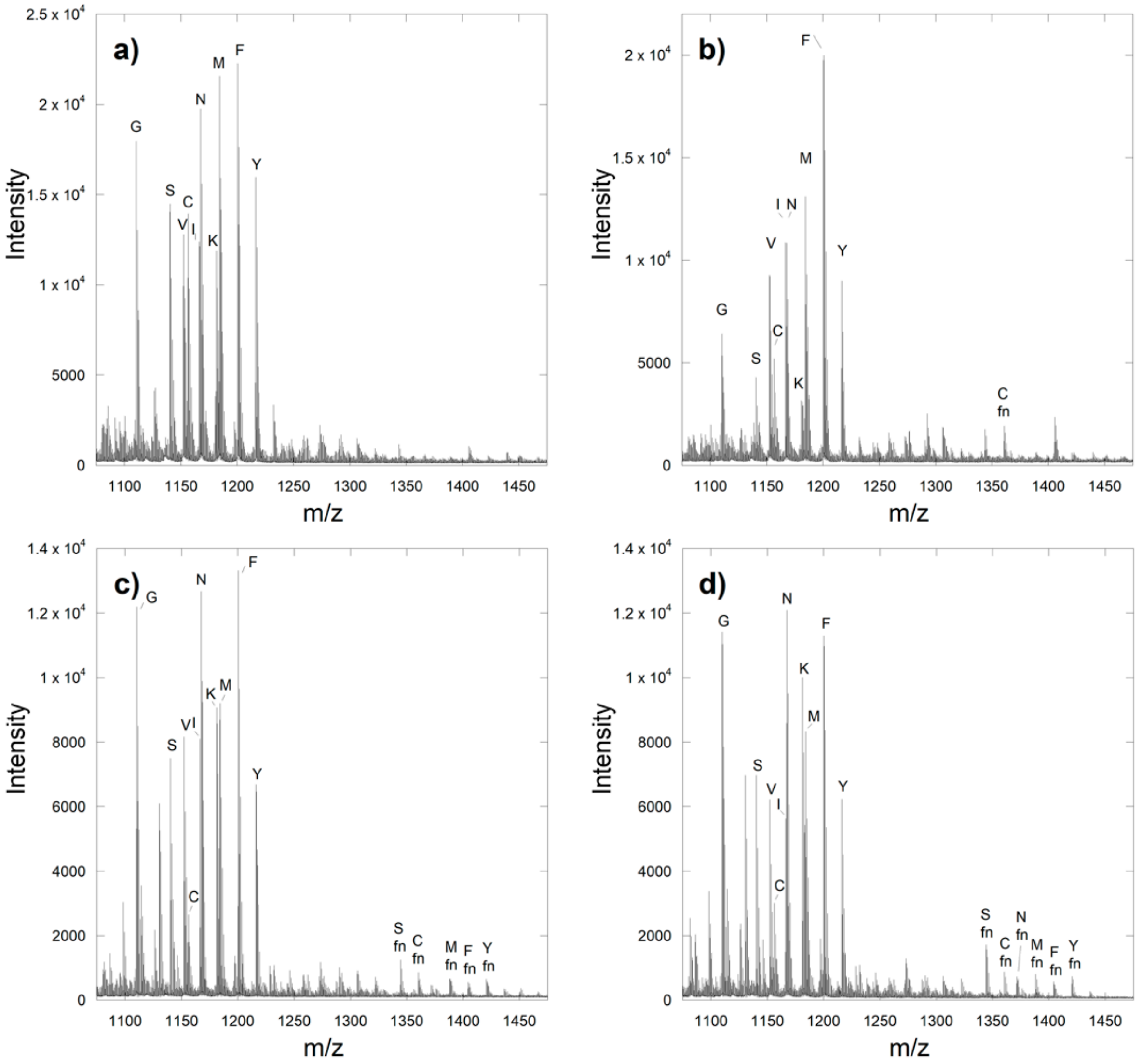

Figure S1. Optimization of enzyme concentration for CaaaX MALDI. DsGRAGCMIIX library 1 reacted with (a) no enzyme (b) 100 nM enzyme (c) 1 uM enzyme (d) 10 uM enzyme

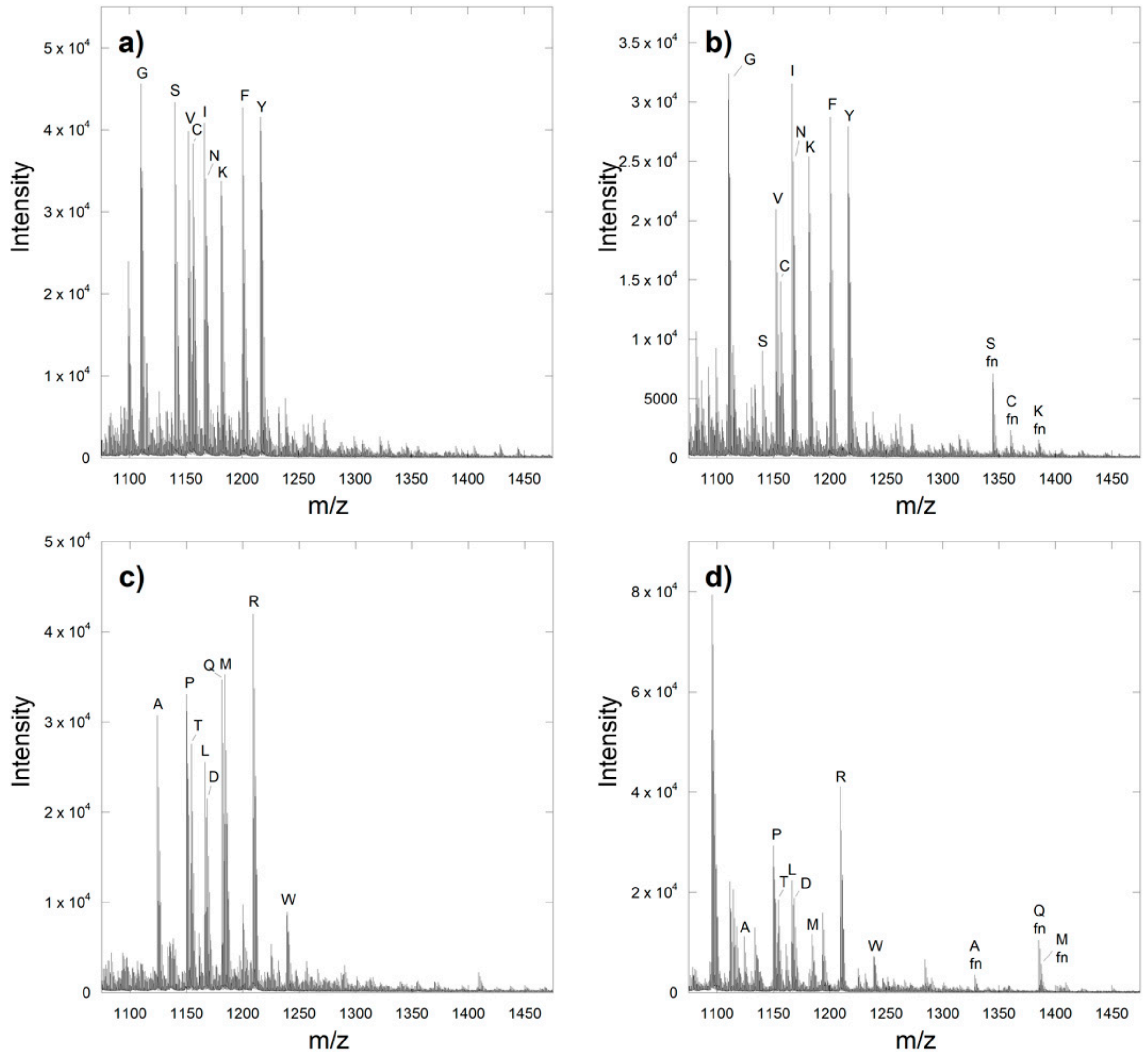

Figure S2. MALDI/MS of DsGRAGCMIIX libraries. Library 1 (a) before and (b) after prenylation with 1 uM yFTase. Library 2 (c) before and (d) after prenylation with 1 uM yFTase.

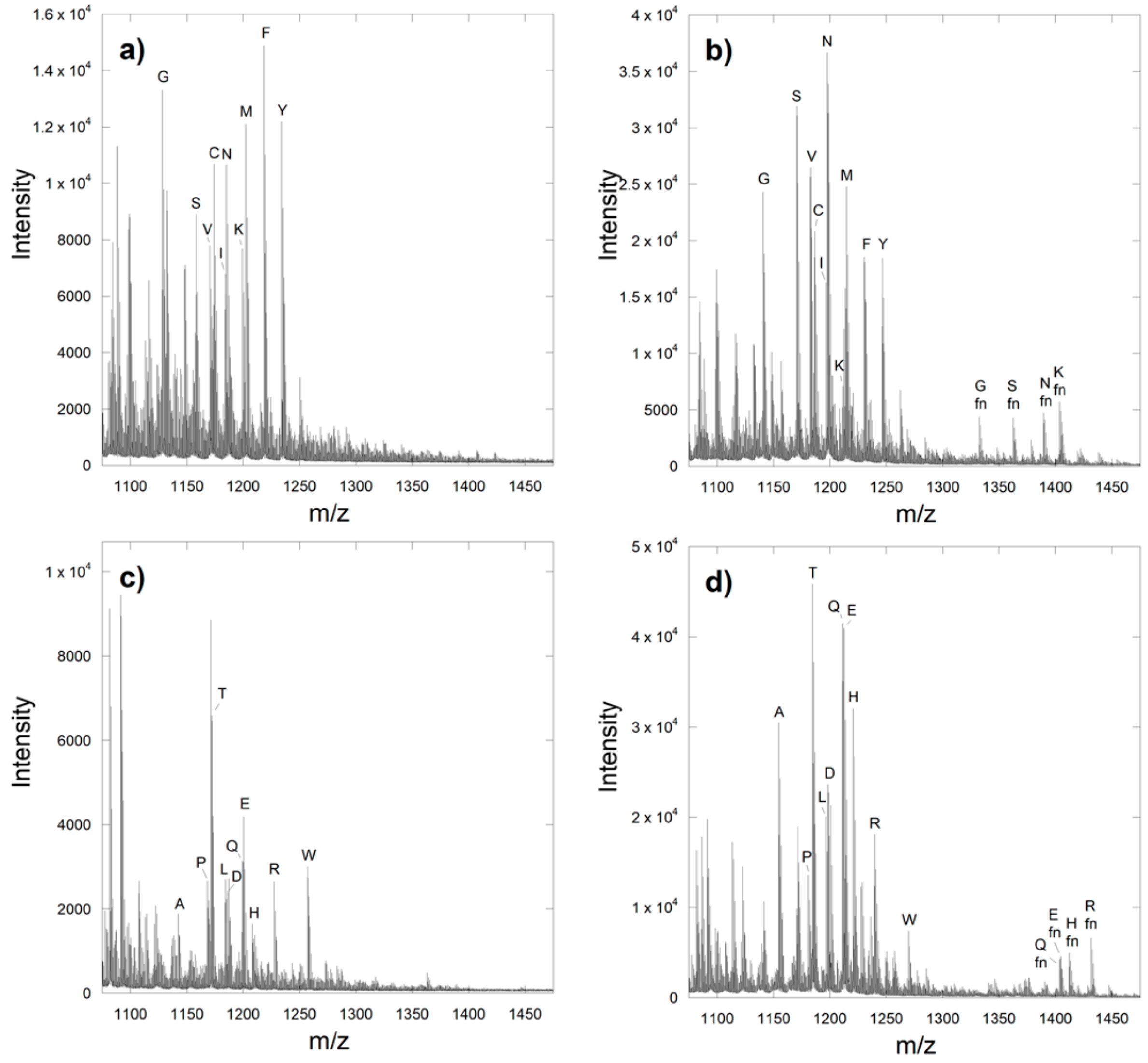

Figure S3. MALDI/MS of DsGRAGCMa$_2$IM libraries. Library 1 (a) before and (b) after prenylation with 1 uM yFTase. Library 2 (c) before and (d) after prenylation with 1 uM yFTase.

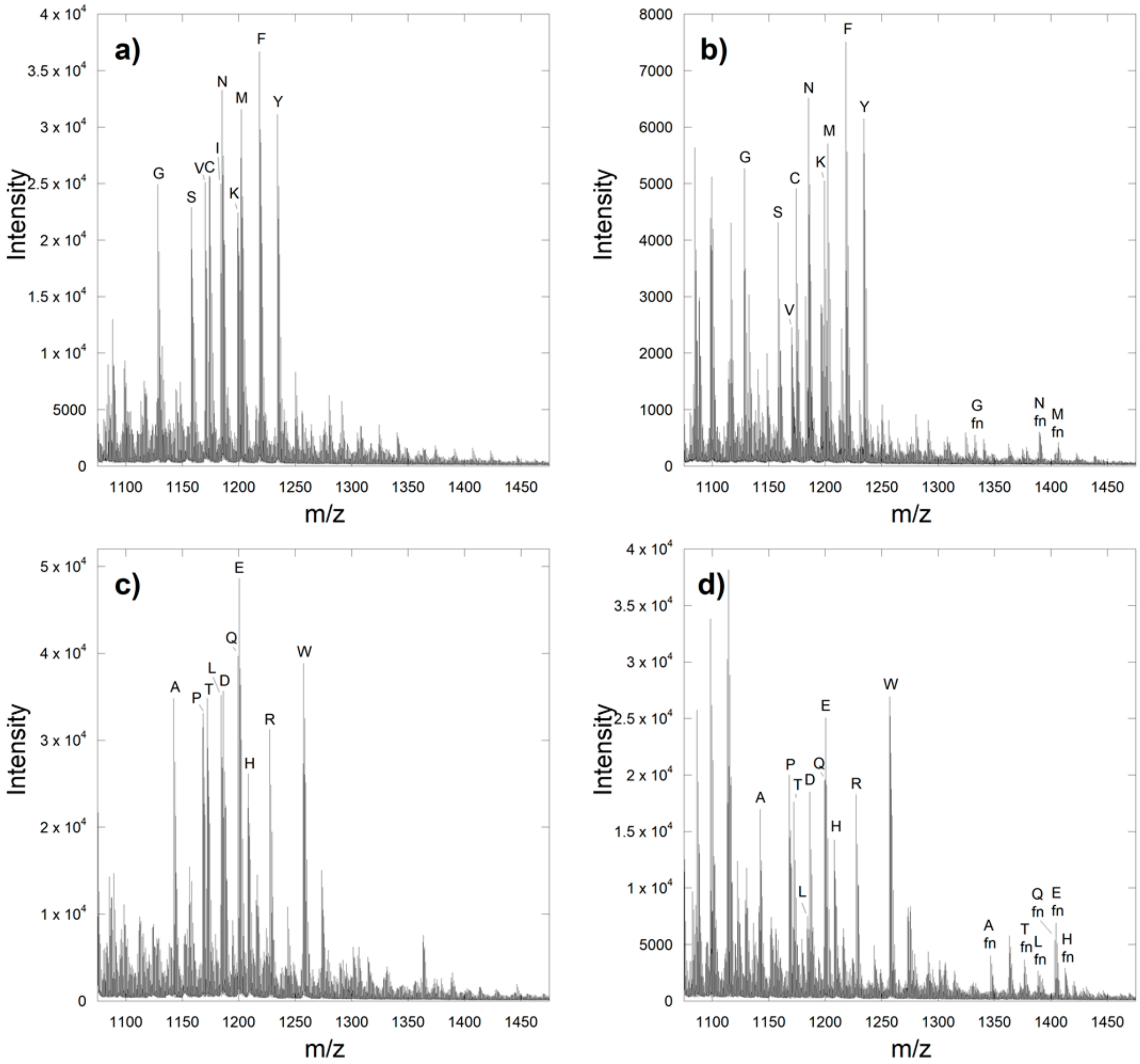

Figure S4. MALDI/MS of DsGRAGCMIa$_3$M libraries. Library 1 (a) before and (b) after prenylation with 1 uM yFTase. Library 2 (c) before and (d) after prenylation with 1 uM yFTase.

Sequence	Formula	+/- Fn	ion	Expected mass	Observed mass
DsGRAGCMIIS	$C_{63}H_{103}N_{13}O_{13}S_{3}2+$	+	M+2H	672.85	672.85
	$C_{25}H_{35}N_{8}O_{6}S+$	+	b4	575.24	575.24
	$C_{43}H_{64}N_{9}O_{7}S_{2}+$	+	b5	882.44	882.43
	$C_{48}H_{73}N_{10}O_{8}S_{3}+$	+	b6	1013.48	1013.48
	$C_{54}H_{84}N_{11}O_{9}S_{3}+$	+	b7	1126.56	1126.56
	$C_{48}H_{78}N_{13}O_{13}S_{3}+$	-	M+H	1140.50	1140.50
	$C_{25}H_{35}N_{8}O_{6}S+$	-	b4	575.24	575.24
	$C_{28}H_{40}N_{9}O_{7}S_{2}+$	-	b5	678.25	678.25
	$C_{33}H_{49}N_{10}O_{8}S_{3}+$	-	b6	809.29	809.29
DsGRAGCMIIQ	$C_{65}H_{106}N_{14}O_{13}S_{3}2+$	+	M+2H	693.36	693.36
	$C_{25}H_{35}N_{8}O_{6}S+$	+	b4	575.24	575.24
	$C_{43}H_{64}N_{9}O_{7}S_{2}+$	+	b5	882.44	882.43
	$C_{48}H_{73}N_{10}O_{8}S_{3}+$	+	b6	1013.48	1013.47
	$C_{54}H_{84}N_{11}O_{9}S_{3}+$	+	b7	1126.56	1126.56
	$C_{60}H_{95}N_{12}O_{10}S_{3}+$	+	b8	1239.65	1239.64
	$C_{50}H_{81}N_{14}O_{13}S_{3}+$	-	M+H	1181.53	1181.53
	$C_{25}H_{35}N_{8}O_{6}S+$	-	b4	575.24	575.24
	$C_{28}H_{40}N_{9}O_{7}S_{2}+$	-	b5	678.25	678.25
	$C_{33}H_{49}N_{10}O_{8}S_{3}+$	-	b6	809.29	809.29
DsGRAGCSIIM	$C_{63}H_{103}N_{13}O_{13}S_{3}2+$	+	M+2H	672.85	672.85
	$C_{25}H_{35}N_{8}O_{6}S+$	+	b4	575.24	575.24
	$C_{43}H_{64}N_{9}O_{7}S_{2}+$	+	b5	882.44	882.43
	$C_{46}H_{69}N_{10}O_{9}S_{2}+$	+	b6	969.47	969.46
	$C_{52}H_{80}N_{11}O_{10}S_{2}+$	+	b7	1082.55	1082.54
	$C_{48}H_{78}N_{14}O_{14}S_{3}+$	-	M+H	1140.50	1140.50
	$C_{25}H_{35}N_{8}O_{6}S+$	-	b4	575.24	575.24
	$C_{28}H_{40}N_{9}O_{7}S_{2}+$	-	b5	678.25	678.25
	$C_{31}H_{45}N_{10}O_{9}S_{2}+$	-	b6	765.28	765.28
DsGRAGCMKIM	$C_{65}H_{108}N_{14}O_{12}S_{4}+$	+	M+2H	702.36	702.36
	$C_{25}H_{35}N_{8}O_{6}S+$	+	b4	575.24	575.24

	$C_{43}H_{64}N_9O_7S_2$+	+	b5	882.44	882.43
	$C_{48}H_{73}N_{10}O_8S_3$+	+	b6	1013.48	1013.48
	$C_{50}H_{83}N_{14}O_{12}S_4$+	-	M+H	1199.53	1199.53
	$C_{25}H_{35}N_8O_6S$+	-	b4	575.24	575.24
	$C_{28}H_{40}N_9O_7S_2$+	-	b5	678.25	678.25
	$C_{33}H_{49}N_{10}O_8S_3$+	-	b6	809.29	809.29
	$C_{39}H_{61}N_{12}O_9S_3$+	-	b7	937.38	937.39
	$C_{45}H_{72}N_{13}O_{10}S_3$+	-	b8	1050.47	1050.47
DsGRAGCYIIM	$C_{69}H_{107}N_{13}O_{13}S_3$2+	+	M+2H	710.86	710.86
	$C_{23}H_{32}N_7O_5S$+	+	b3	518.22	518.22
	$C_{25}H_{35}N_8O_6S$+	+	b4	575.24	575.24
	$C_{43}H_{64}N_9O_7S_2$+	+	b5	882.44	882.44
	$C_{52}H_{73}N_{10}O_9S_2$+	+	b6	1045.50	1045.50
	$C_{58}H_{84}N_{11}O_{10}S_2$+	+	b7	1158.58	1158.58
	$C_{23}H_{33}N_7O_5S$+	-	b3	518.22	518.23
	$C_{25}H_{35}N_8O_6S$+	-	b4	575.24	575.24
	$C_{28}H_{40}N_9O_7S_2$+	-	b5	678.25	678.25
	$C_{37}H_{49}N_{10}O_9S_2$+	-	b6	841.31	841.31
DsGRAGCHIIM	$C_{66}H_{105}N_{15}O_{12}S_3$2+	+	M+2H	697.86	697.86
	$C_{25}H_{35}N_8O_6S$+	+	b4	575.24	575.24
	$C_{43}H_{64}N_9O_7S_2$+	+	b5	882.44	882.43
	$C_{49}H_{71}N_{12}O_8S_2$+	+	b6	1019.50	1019.49
	$C_{51}H_{80}N_{15}O_{12}S_3$+	-	M+H	1190.53	1190.53
	$C_{25}H_{35}N_8O_6S$+	-	b4	575.24	575.24
	$C_{28}H_{40}N_9O_7S_2$+	-	b5	678.25	678.25
	$C_{34}H_{47}N_{12}O_8S_2$+	-	b6	815.31	815.32
DsGRAGCMIGM	$C_{61}H_{99}N_{13}O_{12}S_4$2+	+	M+2H	666.82	666.82
	$C_{25}H_{35}N_8O_6S$+	+	b4	575.24	575.23
	$C_{43}H_{64}N_9O_7S_2$+	+	b5	882.44	882.43
	$C_{48}H_{78}N_{10}O_8S_3$+	+	b6	1013.48	1013.48
	$C_{54}H_{84}N_{11}O_9S_3$+	+	b7	1026.56	1026.58
	$C_{56}H_{87}N_{12}O_{10}S_3$+	+	b8	1183.58	1183.58
	$C_{46}H_{74}N_{13}O_{12}S_4$+	-	M+H	1128.45	1128.45

	$C_{25}H_{35}N_{8}O_{6}S^+$	-	b4	575.24	575.23
	$C_{28}H_{40}N_{9}O_{7}S_{2}^+$	-	b5	678.25	678.25
	$C_{33}H_{49}N_{10}O_{8}S_{3}^+$	-	b6	809.29	809.28

Table S2. MS/MS of prenylated CaaaX library hits. MS/MS ions of prenylated peptides, indicating the Cys is prenylated. b ions for the prenylated peptide are shown, as well as b ions that have also lost the prenyl group.

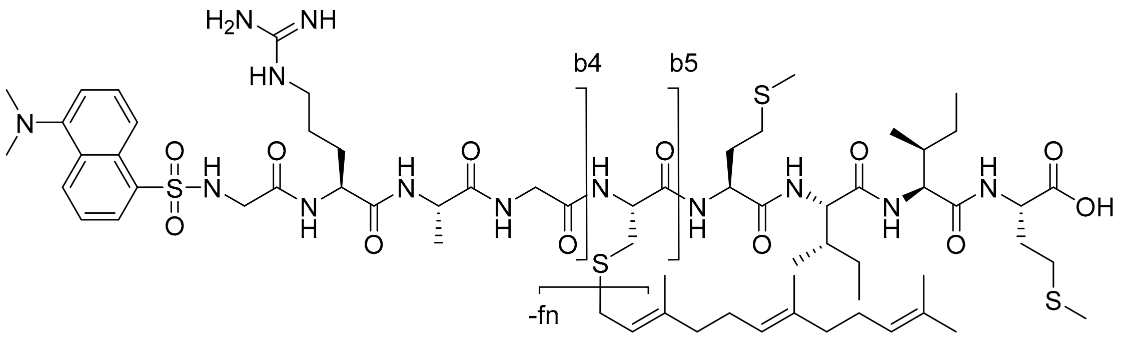

Figure S5. Structure of representative DsGRAGC(fn)MIIM peptide. The major ions used to confirm S-farnesylation are labeled

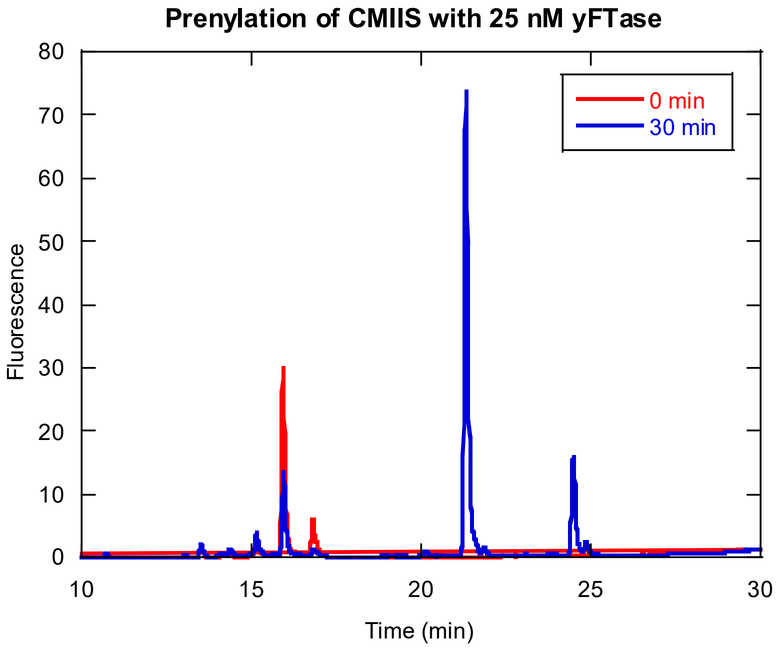

Figure S6. HPLC Fluorescence assay of CMIIS. HPLC assay quantifying the farnesylation of the peptide DsGRAGCMIIS by the fluorescence of the Dansyl group (ex. 220/em. 495). Reaction of 2.4 uM peptide with 25 nM yFTase

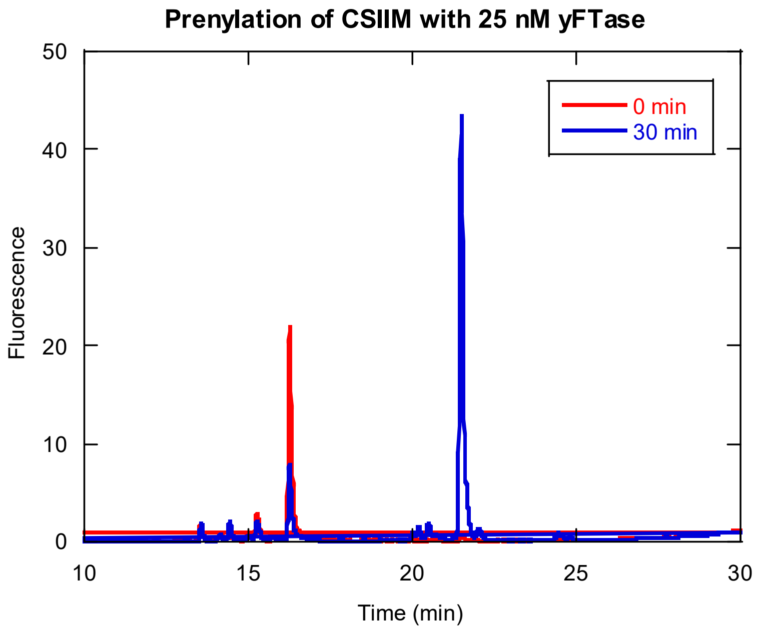

Figure S7. HPLC Fluorescence assay of CSIIM**.** HPLC assay quantifying the farnesylation of the peptide DsGRAGCSIIM by the fluorescence of th Dansyl group (ex. 220/em. 495). Reaction of 2.4 uM peptide with 25 nM yFTase.

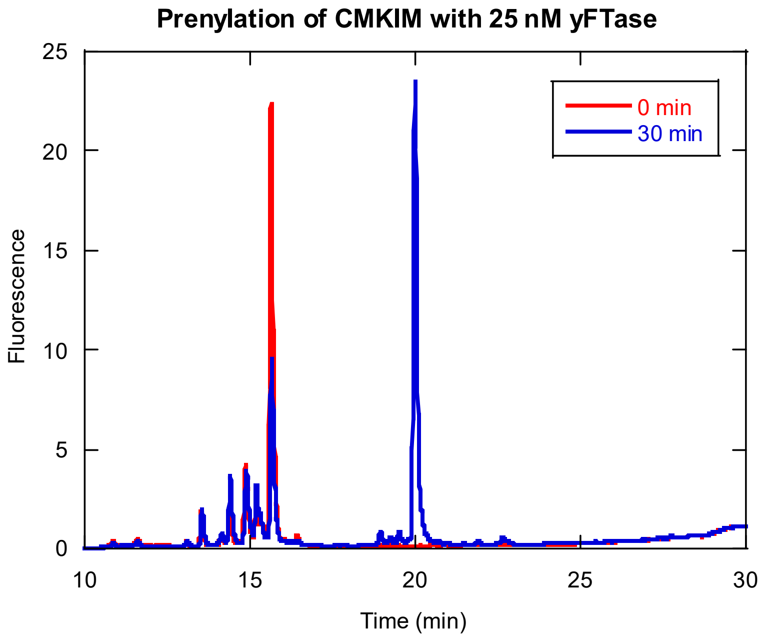

Figure S8. HPLC Fluorescence assay of CMKIM**.** HPLC assay quantifying the conversion the peptide DsGRAGCMKIM by the fluorescence of the Dansyl group (ex. 220/em. 495). Reaction of 2.4 uM peptide with 25 nM yFTase.

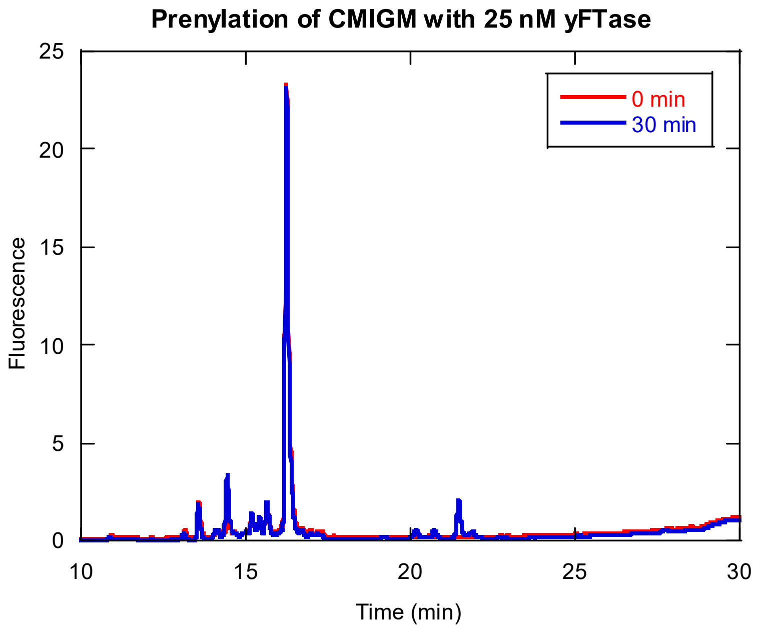

Figure S9. HPLC Fluorescence assay of CMGIM. HPLC assay quantifying the conversion the peptide DsGRAGCMIGM by the fluorescence of the Dansyl group (ex. 220/em. 495). Reaction of 2.4 uM peptide with 25 nM yFTase.

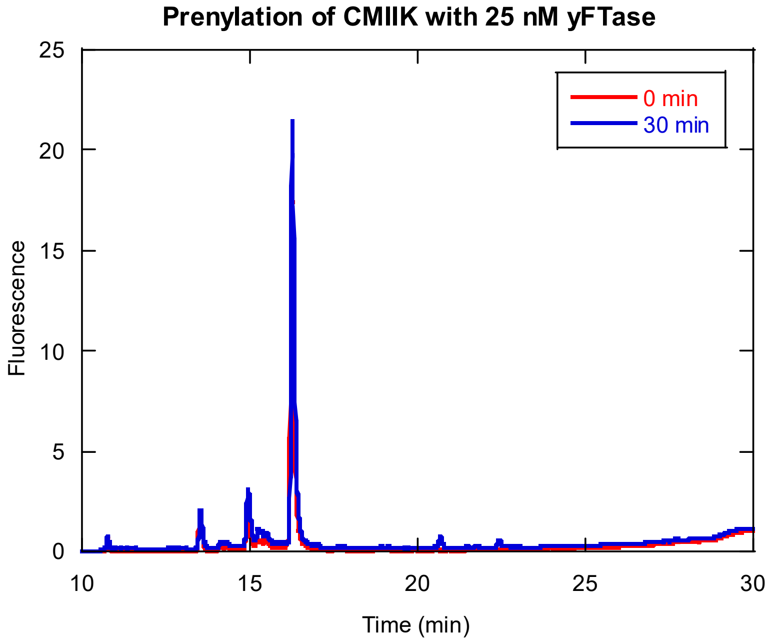

Figure S10. HPLC Fluorescence assay of CMIIK. HPLC assay quantifying the conversion the peptide DsGRAGCMIIK by the fluorescence of Dansyl group (ex. 220/em. 495). Reaction of 2.4 uM peptide with 25 nM yFTase.

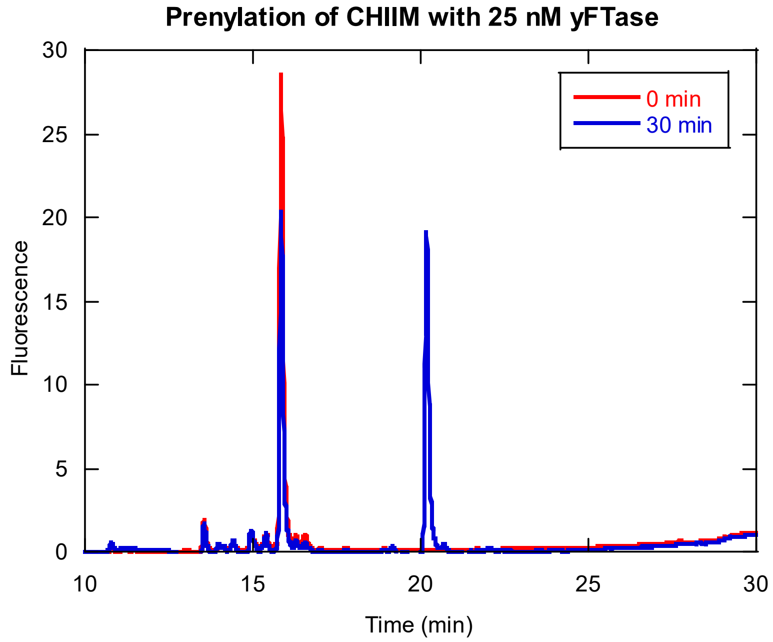

Figure S11. HPLC Fluorescence assay of CHIIM. HPLC assay quantifying the conversion the peptide DsGRAGCHIIM by the fluorescence of Dansylglycine (ex. 220/em. 495). Reaction of 2.4 uM peptide with 25 nM yFTase

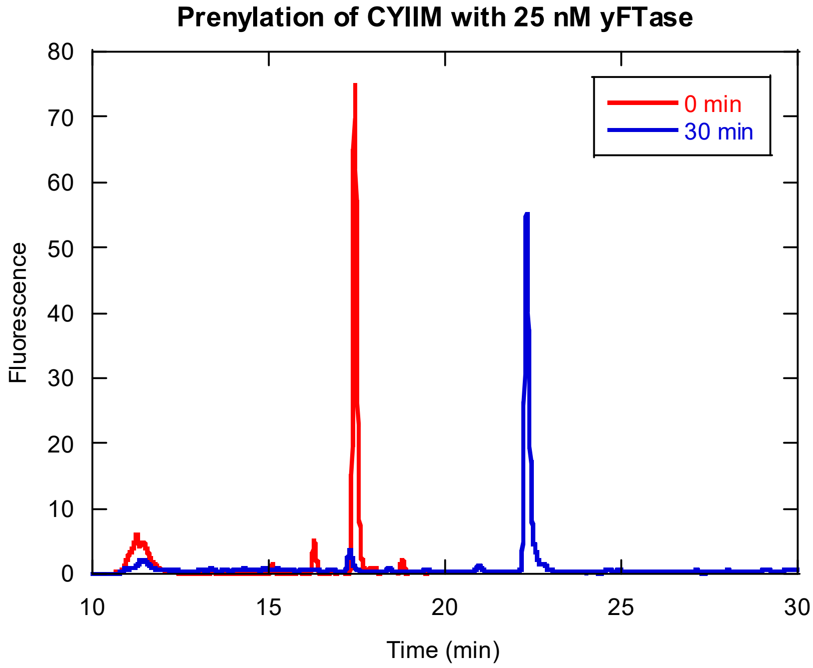

Figure S12. HPLC Fluorescence assay of CYIIM. HPLC assay quantifying the conversion the peptide DsGRAGCYIIM by the fluorescence of Dansyl group (ex. 220/em. 495). Reaction of 2.4 uM peptide with 25 nM yFTase.

Sequence	Protein name	Uniprot ID
CSQAS	Xaa-Pro aminopeptidase 3	https://www.uniprot.org/uniprot/Q9NQH7
CSLMQ	Transcription elongation factor A protein 3	https://www.uniprot.org/uniprot/O75764-2
CSTAN	Neuronal acetylcholine receptor subunit alpha-7	https://www.uniprot.org/uniprot/P36544-3
CASSQ	T cell receptor beta variable 14	https://www.uniprot.org/uniprot/A0A5B0
CMTSQ	Beta-1,4 N-acetylgalactosaminyltransferase 1	https://www.uniprot.org/uniprot/Q00973
CLTIQ	Acetyl-coenzyme A synthetase, cytoplasmic	https://www.uniprot.org/uniprot/Q9NR19
CVQTS	Thymosin beta-15A	https://www.uniprot.org/uniprot/P0CG34
CNVTS	Orphan sodium- and chloride-dependent neurotransmitter transporter NTT5	https://www.uniprot.org/uniprot/Q9GZN6
CASLS	Proline-rich protein 5-like	https://www.uniprot.org/uniprot/Q6MZQ0
CLISS	Regulator of G-protein signaling 7-binding protein	https://www.uniprot.org/uniprot/Q6MZT1
CQLNS	Dynein heavy chain 7	https://www.uniprot.org/uniprot/Q8WXX0
CTASS	Serine/threonine-protein kinase Nek7	https://www.uniprot.org/uniprot/Q8TDX7
CSKLN	NUAK family SNF1-like kinase 1	https://www.uniprot.org/uniprot/O60285
CSKLN	Olfactory receptor 4A8	https://www.uniprot.org/uniprot/P0C604
CSKVN	Thioredoxin domain-containing protein 16	https://www.uniprot.org/uniprot/Q9P2K2
CSLQQ	Uncharacterized protein C1orf109	https://www.uniprot.org/uniprot/Q9NX04-2
CSLLL	Arylsulfatase B	https://www.uniprot.org/uniprot/P15848-2
CSLFA	NF-kappa-B-activating kinase-associated protein 1	https://www.uniprot.org/uniprot/Q9H6S1-4
CSIFI	Nuclear receptor coactivator 7	https://www.uniprot.org/uniprot/Q8NI08-6
CSSAV	Upstream transcription factor 3	https://www.uniprot.org/uniprot/Q68DE3
CSNTF	Protein virilizer homolog	https://www.uniprot.org/uniprot/Q69YN4-4
CAFLS	Histone-lysine N-methyltransferase MECOM	https://www.uniprot.org/uniprot/Q03112-9
CFLSS	N-alpha-acetyltransferase 16, NatA auxiliary subunit	https://www.uniprot.org/uniprot/Q6N069-4
CLLFS	Nuclear mitotic apparatus protein 1	https://www.uniprot.org/uniprot/Q14980-3
CVSVS	Phosphatidylinositol 4-phosphate 5-kinase type-1 gamma	https://www.uniprot.org/uniprot/O60331-2
CQYNS	Pituitary homeobox 1	https://www.uniprot.org/uniprot/P78337
CLFLS	Regulation of nuclear pre-mRNA domain-containing protein 2	https://www.uniprot.org/uniprot/Q5VT52-4
CLACS	Slit homolog 3 protein	https://www.uniprot.org/uniprot/O75094
CASWQ	lamin subunit gamma 3	https://www.uniprot.org/uniprot/Q9Y6N6

Table S3. CaaaX sequences in the human genome. Selected CaaaX sequences in the human genome and their associated proteins. Peptides selected for evaluation are shaded in grey.

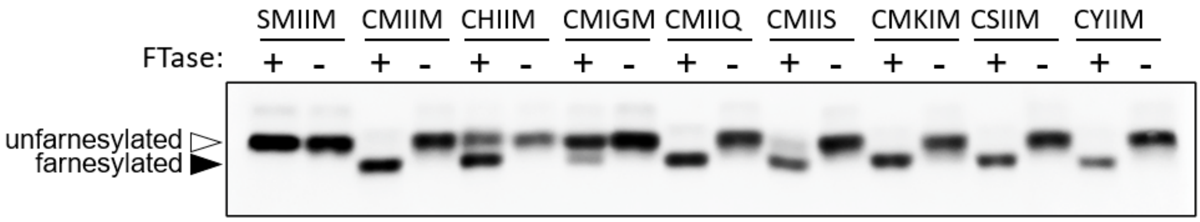

Figure S13. Mobility shift analysis of Ydj1p-CaaaX variants in the presence and absence of farnesyltransferase. Whole cell lysates were prepared and analyzed as described for Figure 5 except that each Ydj1p-CaaaX variant was expressed in two strains. One expresses FTase (yWS2544; *ydj1::KAN*^{R}) and the other lacks FTase (yWS2452; *ydj1::KAN*^{R} *ram1*Δ). The presence (+) or absence of FTase (-) correlates with the presence and absence, respectively, of the non-essential yeast gene RAM1 that encodes the beta subunit of farnesyltransferase.

Sample	Sequence	Biological Replicates	Technical Replicates	Average of Tech. Replicates[1]	Standard Deviation[2]
Controls	CASQ	4	5	100.0%	0.0%
	SASQ	4	5	0.0%	0.0%
	CMIIM	4	7	100.0%	0.0%
	SMIIM	4	9	0.0%	0.0%
Sequences from MALDI libraries	CHIIM	3	4	76.9%	25.9%
	CMIGM	3	5	39.5%	24.8%
	CMIIQ	2	4	100.0%	0.0%
	CMIIS	3	5	88.8%	10.9%
	CMKIM	3	5	99.4%	1.3%
	CSIIM	3	5	100.0%	0.0%
	CYIIM	3	5	100.0%	0.0%
Sequences from the mammalian genome	CASLS	1	3	2.1%	3.6%
	CASSQ	1	3	0.0%	0.0%
	CLACS	1	3	0.6%	1.0%
	CLLFS	1	3	16.4%	2.4%
	CMTSQ	1	3	0.7%	1.2%
	CQYNS	1	3	0.0%	0.0%
	CSKLN	1	3	13.0%	5.5%
	CSLMQ	1	4	96.7%	4.7%
	CSQAS	1	3	0.0%	0.0%
	CVQTS	1	3	1.1%	2.0%

Table S4. Percent farnesylation for Ydj1p-CaaaX variants evaluated in this study.

[1]Values for band intensities of farnesylated and unfarnesylated species within gel lanes of Figures S12 and S13 were determined by the peak integration method using ImageJ [4]. reference; see below for options). These values were used to calculate percent farnesylation for individual samples that were averaged for multiple biological and/or technical replicates.

[2]Standard deviations were derived for all replicates using the Excel STDEV function.

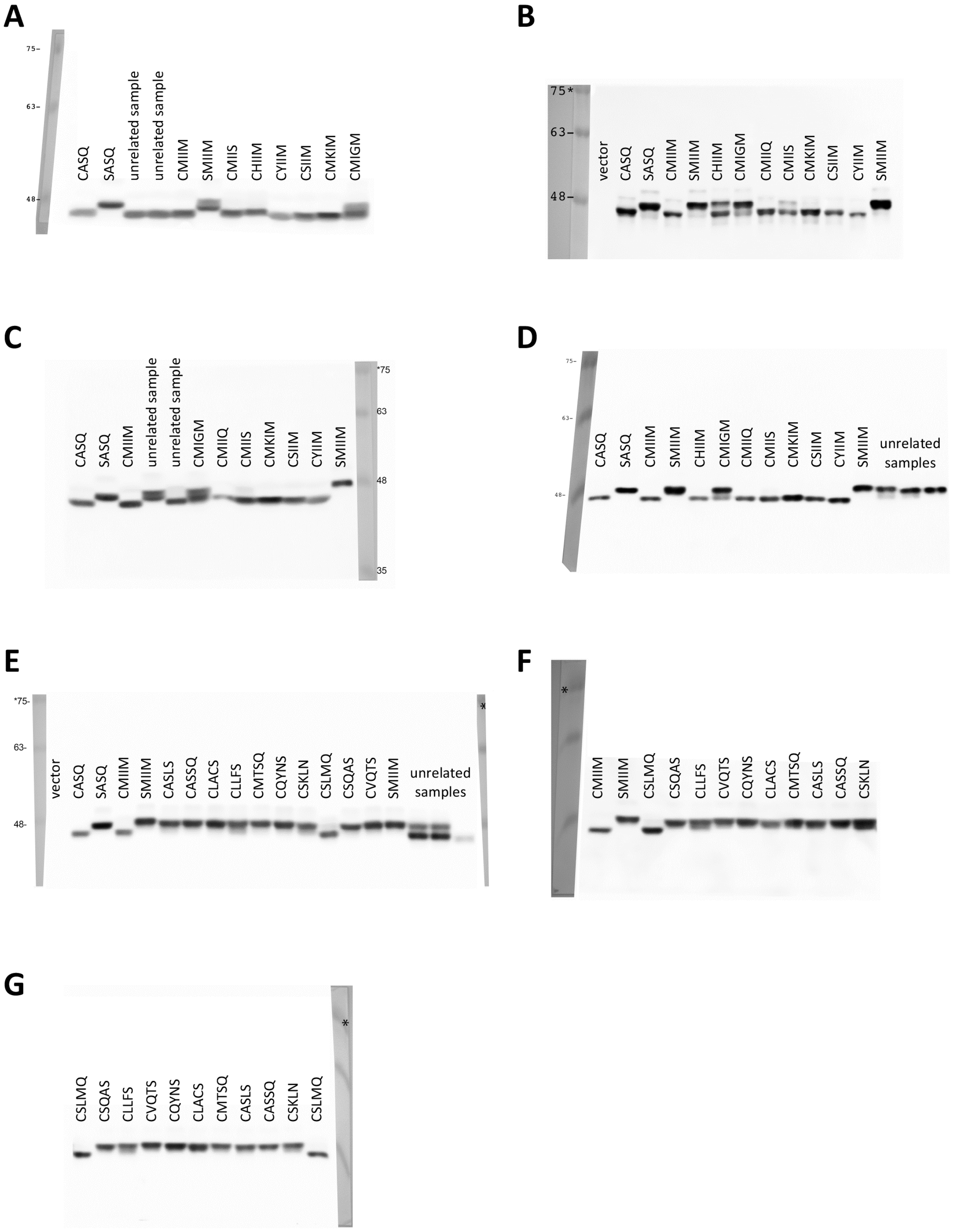

Figure S14. Mobility shift analysis of Ydj1p-CaaaX variants. A collage of anti-Ydj1p immunoblots representing the multiple biological and technical replicates of each Ydj1p-CaaaX variant that was analyzed in this study. Analysis was as described for Figures 5 and 6, with panels D and E representing uncropped versions of the blots used for Figures 5 and 6, respectively. These blots and the blot described for Figure S12 were used for determining the percent prenylation observed for each Ydj1p-CaaaX variant that is reported in Table S6. In each panel, the digital layer containing the anti-Ydj1p chemiluminescence signal is juxtaposed with the layer containing the prestained molecular weight standards that was captured in parallel under ambient light.

Strain	Genotype	Reference
yWS2542	*MAT***a** *his3Δ1 leu2Δ0 met15Δ0 ura3Δ0 ram1::KAN*^{R} *ydj1::NAT*^{R}	[1]
yWS2544	*MAT***a** *his3Δ1 leu2Δ0 met15Δ0 ura3Δ0 ydj1::NAT*^{R}	[1]

Table S5. Yeast strains used in this study.

plasmid	genotype	Reference
pRS416	*CEN URA3* (vector)	[2]
pWS942	*CEN URA3 YDJ1*	[3]
pWS1132	*CEN URA3 YDJ1-SASQ*	[3]
pWS1488	*CEN URA3 YDJ1-CMIIM*	[4]
pWS1917	*CEN URA3 YDJ1-SMIIM*	this study
pWS1918	*CEN URA3 YDJ1-CMIIS*	this study
pWS1919	*CEN URA3 YDJ1-CMIIQ*	this study
pWS1920	*CEN URA3 YDJ1-CHIIM*	this study
pWS1921	*CEN URA3 YDJ1-CYIIM*	this study
pWS1922	*CEN URA3 YDJ1-CSIIM*	this study
pWS1923	*CEN URA3 YDJ1-CMKIM*	this study
pWS1924	*CEN URA3 YDJ1-CMIGM*	this study
pWS1981	*CEN URA3 YDJ1-CSLMQ*	this study
pWS1982	*CEN URA3 YDJ1-CSQAS*	this study
pWS1983	*CEN URA3 YDJ1-CLLFS*	this study
pWS1984	*CEN URA3 YDJ1-CVQTS*	this study
pWS1985	*CEN URA3 YDJ1-CQYNS*	this study
pWS1986	*CEN URA3 YDJ1-CLACS*	this study
pWS1987	*CEN URA3 YDJ1-CMTSQ*	this study
pWS1988	*CEN URA3 YDJ1-CASLS*	this study
pWS1989	*CEN URA3 YDJ1-CASSQ*	this study
pWS1990	*CEN URA3 YDJ1-CSKLN*	this study

Table S6. Plasmids used in this study.

References

[1] S. Ashok, E.R. Hildebrandt, C.S. Ruiz, D.S. Hardgrove, D.W. Coreno, W.K. Schmidt, J.L. Hougland, Protein Farnesyltransferase Catalyzes Unanticipated Farnesylation and Geranylgeranylation of Shortened Target Sequences, Biochemistry. 59 (2020) 1149–1162. https://doi.org/10.1021/acs.biochem.0c00081.

[2] R.S. Sikorski, P. Hieter, A system of shuttle vectors and yeast host strains designed for efficient manipulation of DNA in Saccharomyces cerevisiae., Genetics. 122 (1989) 19–27. https://doi.org/10.1093/genetics/122.1.19.

[3] E.R. Hildebrandt, M. Cheng, P. Zhao, J.H. Kim, L. Wells, W.K. Schmidt, A shunt pathway limits the CaaX processing of Hsp40 Ydj1p and regulates Ydj1p-dependent phenotypes, Elife. 5 (2016) 1–22. https://doi.org/10.7554/eLife.15899.

[4] M.J. Blanden, K.F. Suazo, E.R. Hildebrandt, D.S. Hardgrove, M. Patel, W.P. Saunders, M.D. Distefano, W.K. Schmidt, J.L. Hougland, Efficient farnesylation of an extended C-terminal C(x)3X sequence motif expands the scope of the prenylated proteome, J. Biol. Chem. 293 (2018) 2770–2785. https://doi.org/10.1074/jbc.M117.805770.
